# Non-Invasive Salivary Reproductive Endocrine Profiles in Gentoo Penguins

**DOI:** 10.3390/ani16162622

**Published:** 2026-08-21

**Authors:** Manuel De la Riva-Fraga, Gema Silvan, Carlos Barros-García, Juan Carlos Illera, Belen Crespo, Sara Caceres

**Affiliations:** 1 Department of Animal Physiology, Veterinary Medicine School, Complutense University of Madrid, 28040 Madrid, Spain; madelariva@faunia.es (M.D.l.R.-F.); jcillera@ucm.es (J.C.I.); belencre@ucm.es (B.C.); sacacere@ucm.es (S.C.); 2 Departamento Veterinario, Faunia, 28032 Madrid, Spain; 3 Biology Department, Avanqua-Oceanogràfic S.L., 46013 Valencia, Spain; cbarros@oceanografic.org

**Keywords:** penguins, gentoo penguin, saliva, reproductive endocrinology, steroid hormones, non-invasive monitoring, ovulation

## Abstract

Reproductive endocrine monitoring in penguins is fundamental for improving reproductive management, conservation programs, and welfare assessment, but most studies use serum, which requires handling and blood collection. We hypothesized that saliva, which reflects the free fraction of circulating steroids and can be repeatedly collected with minimal disturbance, would be positively associated with serum concentrations, show hormone-specific reproductive temporal changes, and could be used as an alternative matrix for measuring steroid hormones in female gentoo penguins (*Pygoscelis papua*). Saliva from 21 females was collected every 3 days between 21 days before and 20 days after first egg-laying. Progesterone, 17β-estradiol, estrone sulfate, corticosterone, and testosterone were measured using competitive EIAs. Assay evaluation showed recoveries of 90.89–97.96% and intra- and inter-assay coefficients of variation <8%; paired serum–saliva analyses yielded r^2^ ≥ 0.858 for all hormones. Progesterone peaked in W−3 time window, 17β-estradiol in W−5, and estrone sulfate in W−4, with a decrease around egg-laying. These temporal patterns were consistent with endocrine changes associated with follicular growth, maturation, and oviposition. Saliva therefore appears to be a promising minimally invasive matrix for reproductive endocrine monitoring in female gentoo penguins, with potential future applications in welfare assessment and conservation breeding programs.

## Simple Summary

Breeding programs for penguins and other endangered species are of paramount importance for their preservation, which requires understanding their reproductive cycles. However, most studies are conducted using serum, which requires blood sampling and handling of the animals. This study evaluates saliva as an alternative biological matrix for measuring steroid hormones in female gentoo penguins (*Pygoscelis papua*) during their reproductive cycle. The hormonal profiles obtained revealed significant temporal variations in progesterone, 17β-estradiol, estrone sulfate, corticosterone, and testosterone concentrations that were associated with specific reproductive phases. These results indicate that salivary steroid measurements reflect physiological endocrine changes occurring during the reproductive cycle and support the use of saliva as a minimally invasive matrix for monitoring reproductive status in penguins.

## 1. Introduction

The use of non-invasive methodologies for studying reproductive physiology and endocrinology, health status, and stress responses in wildlife has grown exponentially in recent years [1,2,3]. In the specific case of penguins (Order *Sphenisciformes*, Family *Spheniscidae*), seabirds in elevated trophic positions and associated with areas of high oceanic productivity in the Southern Hemisphere that concentrate phytoplankton and support large biomasses of krill and fish, these studies are especially relevant due to their value as a keystone group and sentinel of health in marine and polar ecosystems, and the danger their populations face [4,5]. Particularly, eleven of the eighteen described species are listed as globally threatened by the International Union for Conservation of Nature [6]. Among the main threats are pollution, food reduction, human pressure, and climate change [7,8,9]. As these species are highly dependent on marine environmental conditions, their populations or reproductive success have declined in different regions, as documented in the case of Magellanic penguins [10]. In addition to the challenges they face, several species have extensive breeding areas that lack protection [8], underscoring the urgent need for tools that enhance knowledge and enable minimally disturbing physiological monitoring. Understanding these physiological and endocrine variables is essential for designing and evaluating breeding and conservation programs, as it allows for the identification of reproductive windows, the anticipation of reproductive failures, and the estimation of the impact of environmental or human pressures, thus facilitating the implementation of evidence-based protection measures for threatened species.

Although the biology, ecology and physiology of several penguin species have been studied in detail [11,12], there are still unknowns that hinder the design, development and application of effective conservation programs and protection measures. Among these unknowns are the endocrinology and reproductive physiology of some of the species and their variability depending on the environmental context [11,12]. Specifically, a recent study has shown greater plasticity of the gentoo penguin *Pygoscelis papua* over the other two species of the genus *Pygoscelis*, *Pygoscelis adeliae* and *Pygoscelis antarcticus*, with phenological advances and differential responses to climate change, and adaptation to environmental changes [13]. At the endocrine level, studies carried out in penguins have documented in detail profiles of gonadal steroid hormones (testosterone, estradiol and progesterone) and LH in both *Eudyptes chrysolophus* and *Pygoscelis papua* [14] and their variation throughout the reproductive cycle as in the case of *Pygoscelis adeliae* and *Aptenodytes forsteri* [15], or more recently in *Spheniscus magellanicus* [16], highlighting that reproductive physiology and endocrinology are species- and phase-dependent, producing variations in hormone levels depending on the species and predictable changes between the different phases of the reproductive cycle (courtship, pre-laying/laying and incubation).

Most studies on penguin reproductive physiology have traditionally relied on plasma and serum as biological matrices for hormonal assessment. Nevertheless, blood collection is an invasive procedure that necessitates animal capture, handling, and restraint, all of which may elicit physiological stress responses. The activation of the hypothalamic–pituitary–adrenal axis (HPA) associated with these procedures can rapidly alter circulating hormone concentrations, thereby introducing a potential source of bias in endocrine measurements. Evidence from Adélie penguins and emperor penguins have demonstrated significant increases in corticosterone concentrations associated with handling intensity and restraint duration. Therefore, the use of blood-derived samples may compromise both animal welfare and the accurate characterization of physiological endocrine status, highlighting the need for less invasive monitoring approaches [17]. Recently, in another study carried out on the three species of the genus *Pygoscelis*, it has been described that corticosterone levels are dependent on the species and age of the animals, with chicks being more sensitive than adults [18]. The use of non-invasive matrices such as feces has been employed in several penguin species, such as in the case of *Spheniscus humboldti* for the determination of glucocorticoids, allowing analysis of how environmental effects and stressful events influence physiological processes such as molting or the impact of tourism, and the well-being of these species [19,20,21,22]. But in general, non-invasive matrices do not allow for the collection of instantaneous information, obtaining accumulated information from hours and even days and can be affected by both individual factors such as intestinal transit or external factors such as the type of diet [20]. Other non-invasive matrices used in some bird species have been feathers, used for the study of corticosterone [23,24,25,26]. However, they have the drawback of not collecting acute events, since the information is obtained during their development and in the specific case of penguins it is limited by the annual molting process characteristic of this taxon [27,28]. Consequently, these matrices are less suitable for examining within-individual endocrine changes around discrete reproductive events such as egg-laying.

For this reason, saliva has become particularly important among non-invasive matrices, as it reflects the free fraction of steroid hormones, allows repeated sampling with minimal disturbance from trained or desensitized animals. Compared with feces and feathers, saliva may provide endocrine information closer to the time of sampling and facilitate longitudinal monitoring. Furthermore, its preservation is simpler than that of other biological matrices [29]. In some mammals, it has been used for cortisol measurements, including in aquatic mammals such as manatees and dolphins [30], as well as for monitoring reproductive cycles in manatees and elephants [31,32]. However, in birds, salivary endocrine monitoring remains scarcely explored and has mainly focused on measurement of corticosterone and its correlation with plasma levels, with validations reported in large-billed crows and seagulls [33,34]. To our knowledge, saliva has not previously been evaluated for the longitudinal monitoring of multiple reproductive steroid hormones in penguins. The present study addresses this methodological and conceptual gap.

In this context, the gentoo penguin (*P. papua*) is a particularly suitable model because the species is widely maintained in zoological institutions and has a long history of managed breeding and individual reproductive monitoring. In managed populations its reproductive seasonality is well characterized, breeding histories and first egg-laying dates can be accurately recorded, and repeated voluntary saliva collection is feasible under human care. Following this principle, the objective of this study is to evaluate whether saliva serves as a valid matrix for measuring steroid hormones in female gentoo penguins and to characterize their longitudinal salivary profiles around first egg-laying in order to more accurately discern key events such as ovulation. We hypothesized that salivary steroid concentrations would be positively associated with paired serum concentrations and would show hormone-specific temporal changes consistent with follicular development, maturation, and oviposition.

## 2. Materials and Methods

### 2.1. Animals

In this study saliva samples from 29 female gentoo penguins (*Pygoscelis papua*) belonging to a colony of 89 individuals were collected. The sample size was established based on predefined biological inclusion criteria and following the resource-equation approach described by Festing [35]. All females were in reproductive age, ranging from 6 to 23 years, captive-born, and had stable mates for several years. Furthermore, all had laid eggs in the seasons prior to the study.

Of the 29 animals selected, samples from only 21 were used. The exclusion of the other 8 was due to the absence of egg-laying during the study period, which made it impossible to confirm successful ovulation.

The animals were housed in the penguinarium at Faunia (Madrid, Spain). It was an indoor facility with a land area of approximately 400 m^2^ and a pool with a volume of 500 m^3^ and a maximum depth of 4.5 m. The enclosure was shared with five other penguin species (king *Aptenodytes patagonicus*, Adélie *Pygoscelis adeliae*, macaroni *Eudyptes chrysolophus*, chinstrap *Pygoscelis antarcticus* and southern rockhopper *Eudyptes chrysocome*). Environmental conditions were maintained in accordance with the standards recommended in the AZA Best Practice Guidelines [36]. This included an artificial photoperiod using LED lighting that varied between 5 and 19 h depending on the time of year, an air temperature between −1.5 and 6 °C, and a water temperature between 10 and 14 °C. In addition, environmental modifications were made according to the season, such as the presence of artificial snow between October and March, the subsequent thaw, and the gradual addition of rocks from March to September, coinciding with the breeding season.

The diet consisted of a combination of two types of herring, capelin and sprat, supplemented with mussel, shrimp, and squid. All animals received a daily multivitamin supplement (Akwavit^®^) (Kasper Faunafood, manufactured by Arie Blok Pet Food, Woerden, The Netherlands), administered at a rate of one-quarter of a tablet per animal.

### 2.2. Sample Collection

In this study, a total of 263 saliva samples were collected and analyzed from the 21 included females, during two consecutive breeding seasons, from April to June of 2023 and 2024. The start of the breeding period was previously determined by monitoring environmental factors that induce the reproductive cycle of this species (photoperiod, temperature and environmental changes such as thawing and the introduction of rocks for nest building) and by monitoring characteristic reproductive behavior, including pair establishment and vocalizations, as well as nest building and defense [37,38,39].

Saliva samples were collected every 3 days throughout the study period. Collection was performed using sterile cotton-tipped swabs with plastic shafts (Deltalab, Barcelona, Spain), taking advantage of the animals’ natural beak opening when they asked for food. The swab was kept in the oral cavity for a minimum of 5 s to ensure adequate saturation. The samples were stored at −20 °C until processing.

All saliva samples were collected during the first feeding of the morning, but before food intake, to avoid contamination and minimize variability associated with the circadian rhythm. No regurgitation was observed during sampling, and no visible food particles or regurgitated material were detected on the swabs or in the recovered samples. Animal handling was avoided during sample collection thanks to desensitization.

To validate the hormonal measurement in saliva, blood samples were also obtained from eight females in the study group. Because blood collection required restraint and jugular venipuncture, the number of females included in this complementary validation component was limited in accordance with the reduction principle of the 3Rs and the recommendations of the institutional ethics committee. These samples were taken simultaneously with the saliva samples from the same individuals, allowing for a comparison between the two biological matrices. Blood was drawn from the right jugular vein, considered one of the main venous access routes in birds, following the recommendations previously described for penguins [40]. A 3 mL syringe (B. Braun, Melsungen, Germany) with a 23G needle (Covetrus, Barcelona, Spain) was used for the extraction.

All handling procedures were supervised and approved by Faunia’s animal welfare committee, as well as by an external ethics committee from Oceanografic of Valencia (OCE-29-22).

### 2.3. Sample Processing

Saliva samples were rehydrated with distilled water at a ratio of 1:25 and then centrifuged at 2000× *g* for 20 min at 20 °C. The resulting supernatant was transferred to Eppendorf tubes and frozen at −20 °C until analysis.

Blood samples were collected in Eppendorf tubes and centrifuged at 1200× *g* for 20 min at 4 °C within 2 h of collection. The resulting serum was separated and stored frozen at −20 °C until analysis.

### 2.4. Hormonal Analysis

The concentrations of estrone sulphate (E1S), 17β-estradiol (E2), corticosterone (CT), testosterone (T) and progesterone (P4) were determined by a competitive enzyme immunoassay (EIA), previously developed and validated at the Endocrinology Laboratory of the Department of Animal Physiology of the Faculty of Veterinary Medicine of the Complutense University of Madrid [41].

For this purpose, 96-well flat-bottom polystyrene microtiter plates were used, to which the purified antibody solution of estrone sulphate (E1SO4: R522-2), 17β-estradiol (E2: C6E91), corticosterone (CT: C9134), testosterone (T: R156) and progesterone (P4: C914) was added and incubated at 4 °C overnight.

After this time, the competition reaction was carried out by diluting samples and standards in conjugate working solutions conjugated and added to the coated plates. The conjugates were used at working dilutions of 1:40,000 for corticosterone, progesterone and 17β-estradiol, 1:120,000 for estrone sulfate, and 1:80,000 for testosterone. All samples were determined in duplicate.

After 2 h conjugate incubation at 20 °C, the plates were washed and Enhanced K-Blue^®^ TMB Substrate (Neogen, Lexington, KT, USA) was added to all wells. The colorimetric reaction was stopped by adding a 10% sulfuric acid solution. Absorbance was measured at 450 nm using an automated ELISA reader (Eurogenetics NV, Tessenderlo, Belgium).

Hormone concentrations were calculated using specialized software developed by the Computer Science Department at the University of California, Davis. For each hormone, a response curve was generated plotting the percentage of binding (B/B0 × 100) against the known concentrations of the added hormone standards. Hormone concentrations were expressed in ng/mL for progesterone, corticosterone and testosterone, and in pg/mL for estrone sulphate and 17β-estradiol, and presented as mean ± SD.

### 2.5. Validation Parameters

The validation of the EIA technique for penguin saliva samples was based on accuracy, precision and parallelism between serum and saliva observations obtained from eight females, as summarized in Table 1. The sensitivity of the amplified EIA was tested by means of low detection limit, as defined by Abraham, Munro and Lasley and calculated from B0 defined as the maximum-binding absorbance obtained from the zero-concentration standard minus two standard deviations (2SD) in 10 consecutive assays, and it was: P4 = 13.21 pg/well, T = 11.43 pg/well, E2 = 1.99 pg/well, E1S = 4.54 pg/well, and CT = 5.18 pg/well [42,43]. In order to determine the interferences of saliva on a standard curve, the standard curves in saliva samples were run in parallel with the standard dose–response curve. There was a good degree of parallelism between both standard curves for the hormones studied: P4 r2 = 0.87, E1S r2 = 0.85, E2 r2 = 0.89, T r2 = 0.92 and CT r2 = 0.85. The accuracy of the EIAs was assessed using spike-and-recovery tests with progesterone (P4), testosterone (T), 17β-estradiol (E2), estrone sulfate (E1S), and corticosterone (CT). For each hormone, known amounts of 1, 10, 100, and 500 pg were added separately to 100 µL aliquots of saliva samples representing low and high endogenous hormone concentrations. A corresponding unspiked aliquot of each saliva sample was analyzed in parallel to determine its endogenous hormone concentration. Recovery rates were: P4 = 96.14 ± 7.18; T = 90.89 ± 9.36; E2 = 97.96 ± 4.98; E1S = 91.20 ± 3.45; and CT = 92.05 ± 9.26. Precision was determined by calculating the intra- and inter-assay coefficients of variation (CV%). The intra-assay CV (%) was calculated by replicate measurements of known saliva sample. Each sample was tested in triplicate 10 times within an assay. Intra-assay percentages were as follows: P4 = 7.08 ± 1.62; T = 3.07 ± 0.25; E2 = 7.07 ± 0.56; E1S = 7.54 ± 1.04; and CT = 5.36 ± 0.42. Inter-assay CV (%) was calculated by the replicate measurements of a known saliva sample in 10 consecutive assays. Inter-assay results were: P4 = 7.48 ± 0.26; T = 6.54 ± 1.17; E2 = 7.91 ± 0.22; E1S = 6.83 ± 0.55; and CT = 6.76 ± 0.71. In order to validate the results from saliva samples, the correlation between serum and saliva samples obtained at the same time from eight females were analyzed using Pearson’s correlation. Pearson correlations and the corresponding coefficients of determination showed positive associations between serum and saliva concentrations for all hormones: P4, r^2^ = 0.910, *p* < 0.001; E1S, r^2^ = 0.858, *p* < 0.001; E2, r^2^ = 0.879, *p* < 0.001; T, r^2^ = 0.990, *p* < 0.001; and CT, r^2^ = 0.919, *p* < 0.001.

### 2.6. Statistical Analysis

Statistical analyses were performed using IBM SPSS Statistics, 29.0 (IBM Corp., Chicago, IL, USA). The temporal variation in salivary hormone concentrations was evaluated using linear mixed models, with female identity included as a random intercept to account for repeated observations within individuals. For models treating day relative to first egg-laying as a continuous covariate, female-specific random slopes were also evaluated using unstructured and diagonal covariance structures. However, these models failed to converge reliably and produced boundary or redundant slope-variance estimates across hormones. Therefore, the final models retained female identity as a random intercept only. Two complementary temporal approaches were considered day relative to laying as continuous covariate, taking day 0 as the date of laying the first egg, and reproductive window as categorical fixed effect.

For the comparison of hormonal values, the sampling days were grouped into 3−day windows, from 21 days before laying (day −21) to 20 days after laying (day +20). This grouping allowed us to reduce the variability associated with non-daily sampling and standardize the temporal comparison between individuals. The windows defined based on that grouping were W−7 (−21 to −19), W−6 (−18 to −16), W−5 (−15 to −13), W−4 (−12 to −10), W−3 (−9 to −7), W−2 (−6 to −4), W−1 (−3 to −1), W0 (0 to +2), W+1 (+3 to +5), W+2 (+6 to +8), W+3 (+9 to +11), W+4 (+12 to +14), W+5 (+15 to +17) and W+6 (+18 to +20). Based on these windows, the following reproductive phases were defined:

- Pre-laying: windows W−7 to W−2 (−21 to −4 days);
- Peri-laying: window W−1 (−3 to −1 days);
- Egg-laying: window W0 (0 to +2 days);
- Post-laying: windows W+1 onwards (≥+3 days).

Descriptive results are presented in the original scale as mean ± SD. The need for transformation was evaluated by examining the distribution of each response variable and the residual diagnostics of models fitted on the original scale. Normality and homoscedasticity were assessed using histograms and Q–Q plots of the standardized residuals, together with plots of standardized residuals against fitted values. A natural logarithmic transformation was applied when the original-scale model showed marked right-skewness residual distributions and/or heteroscedasticity and was retained when it improved these diagnostic patterns. These criteria were met for P4, E1S, E2, CT, and T; therefore, inferential analyses for all five hormones were conducted using ln-transformed concentrations.

Although sample collection followed a standardized schedule, the final analyte-specific datasets were unbalanced because the number of valid observations was not identical across reproductive windows. Fixed effects were evaluated using Type III F tests to provide a consistent omnibus assessment across these datasets. Because each model contained only one temporal predictor at a time and no interaction terms, the Type III test represented the corresponding overall temporal effect and was not affected by the order of term entry. When a significant window effect was detected, pairwise comparisons between estimated marginal means were performed using Sidak adjustment for multiple comparisons.

For models fitted using ln-transformed concentrations, estimated marginal means and their 95% confidence intervals (95% CI) were back-transformed to the original scale and are reported as geometric means. Pairwise differences between windows were also back-transformed and expressed as ratios. Differences were considered statistically significant at *p* < 0.05.

## 3. Results

### 3.1. Temporal Variation in Salivary Progesterone and Estrogens (Estradiol and Estrone Sulfate) as Markers of the Reproductive Cycle

#### 3.1.1. Progesterone (P4)

**Figure 1:**
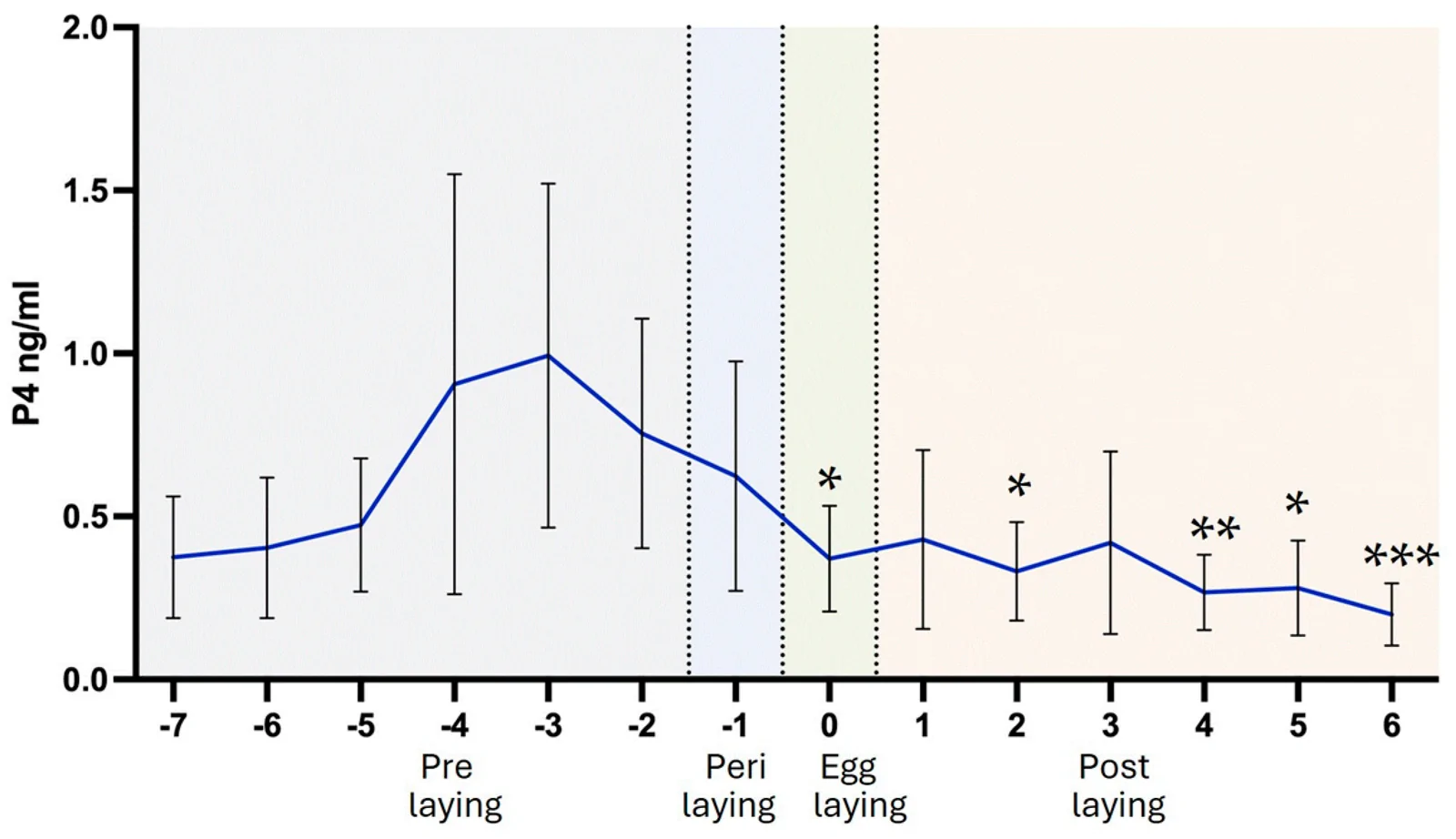
Temporal variation in salivary P4 in female gentoo penguins (*Pygoscelis papua*) during the peri-laying period. Data are presented as mean ± SD for consecutive 3-day reproductive windows from W−7 to W+6, corresponding to 21 days before to 20 days after first egg-laying. Day 0 represents the date of first egg-laying. Reproductive phases are indicated as pre-laying, peri-laying, egg-laying, and post-laying. Asterisks indicate significant Sidak-adjusted comparisons against W−3, the window with the highest progesterone concentration. * *p* < 0.05; ** *p* < 0.01; *** *p* < 0.001. Sample sizes by reproductive window were: W−7, *n* = 18; W−6, *n* = 19; W−5, *n* = 20; W−4, *n* = 19; W−3, *n* = 20; W−2, *n* = 17; W−1, *n* = 19; W0, *n* = 21; W+1, *n* = 18; W+2, *n* = 20; W+3, *n* = 17; W+4, *n* = 18; W+5, *n* = 19; and W+6, *n* = 18 (total *n* = 263).

The temporal pattern of salivary P4 concentrations, characterized by a pre-laying peak followed by a decline around egg-laying, is shown in Figure 1. Salivary P4 showed a marked temporal pattern with an increase during the pre-laying phase, reaching its maximum in window W−3 (days −9 to −7), and a decrease during laying (W0), remaining lower during the post-laying period. In the observed and analyzed data corresponding to the period between W−7 and W+6 (−21 to 20 days), progesterone concentrations averaged 0.523 ± 0.518 ng/mL. The highest individual value was reached on day −8 (W−3). At the window level, the highest concentration was also observed in W−3 with values of 0.993 ± 0.528 ng/mL, while in the window corresponding to the laying W0 (days 0 to +2) the concentrations decreased to 0.370 ± 0.162 ng/mL.

The analysis using linear mixed models employed logarithmically transformed (ln-transform) concentrations and, considering the individual as a random effect, demonstrated the presence of significant decline during the time analyzed (Type III test of fixed effects: *p* < 0.001). When time was grouped into consecutive 3-day windows significant differences between windows were also detected (Type III test of fixed effects: *p* < 0.001).

Back-transformed estimated marginal means showed the highest concentration in W−3 (0.679 ng/mL), followed by a progressive decrease towards W0 (0.290 ng/mL) and the post-laying phase. In pairwise comparisons with Sidak adjustment, the W−3 window was significantly higher than W0 (ratio = 2.34; *p* = 0.020), as well as than several post-laying windows (W+2: ratio = 2.89; *p* = 0.031; W+4: ratio = 3.69; *p* = 0.007; W+5 ratio = 3.45; *p* = 0.026; W+6: ratio = 5.61; *p* < 0.001), confirming a significant decrease in progesterone from the pre-laying peak to laying and the post-laying period.

#### 3.1.2. Estrogens

##### Estrone Sulfate (E1S)

**Figure 2:**
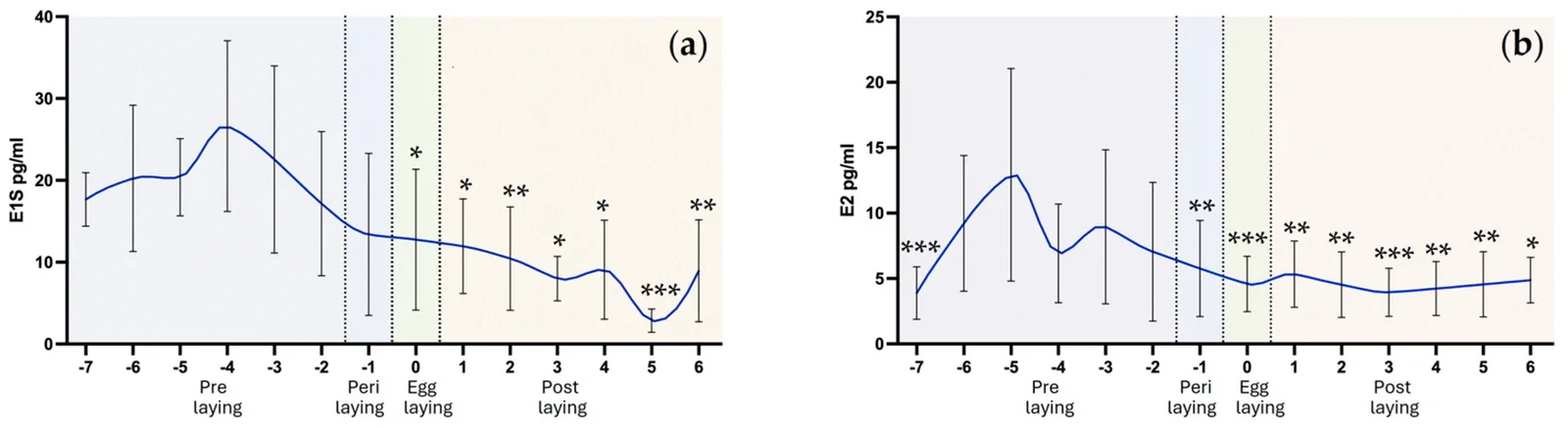
Temporal variation in salivary estrogens in female gentoo penguins (*Pygoscelis papua*) during the reproductive period. For both panels, sample sizes by reproductive window were: W−7, *n* = 18; W−6, *n* = 19; W−5, *n* = 20; W−4, *n* = 19; W−3, *n* = 20; W−2, *n* = 17; W−1, *n* = 19; W0, *n* = 21; W+1, *n* = 18; W+2, *n* = 20; W+3, *n* = 17; W+4, *n* = 18; W+5, *n* = 19; and W+6, *n* = 18 (total *n* = 263). (**a**) E1S concentrations across consecutive 3-day reproductive windows. Asterisks indicate significant Sidak-adjusted comparisons against W−4, the window with the highest E1S concentration. (**b**) E2 concentrations across the same period. Asterisks indicate significant Sidak-adjusted comparisons against W−5, the window with the highest E2 concentration. * *p* < 0.05; ** *p* < 0.01; *** *p* < 0.001. Data are presented as mean ± SD. Day 0 represents first egg-laying.

The temporal profile of salivary E1S across reproductive windows is shown in Figure 2a. In the case of salivary E1S, a clear temporal variation was observed across the analyzed period (W−7 to W+6) with higher concentrations during the pre-laying phase and a marked decrease from laying onwards. The highest individual value was recorded on day −12 (window W−4), whereas the lowest value was observed on day +15 (window W+5). At the window level, the highest mean concentration was observed in W−4 (26.6 ± 10.4 pg/mL), whereas the lowest mean was observed in W+5 (2.85 ± 1.43 pg/mL) followed by a rebound in W+6 (mean 8.96 ± 6.23 pg/mL).

Linear mixed models using ln-transformed concentrations and a random intercept for individual confirmed a significant temporal effect when day relative to laying was included as a continuous covariate (Type III test of fixed effects: *p* < 0.001) and also a significant overall effect when time was grouped into consecutive 3-day windows (Type III test of fixed effects: *p* < 0.001). Sidak-adjusted pairwise comparisons showed that W−4 was significantly higher than W0 (*p* = 0.002), W+1 (*p* = 0.021), W+2 (*p* = 0.005), W+3 (*p* = 0.011), W+4 (*p* = 0.035), W+5 (*p* < 0.001), and W+6 (*p* = 0.003). The strongest contrast was observed between W−4 and W+5, with E1S concentrations being approximately 11.7-fold higher in W−4. In addition, W−3 was significantly higher than W0 (*p* = 0.045), W+5 (*p* < 0.001), and W+6 (*p* = 0.033). These results suggest a decline in E1S from the mid pre-laying to laying and beyond.

##### Estradiol (E2)

The corresponding salivary E2 profile is shown in Figure 2b. Salivary E2 also showed temporal variation during the analyzed period, with the average maximum in the pre-laying phase and lower values around and after laying, with the highest individual E2 concentration was recorded on day −14, within the pre-laying phase. At the window level, W−5 (−15 to −13 days) showed the highest mean values (12.9 ± 8.1 pg/mL), while in the laying phase W0, the values decreased (4.59 ± 2.12 pg/mL).

The linear mixed model showed a significant negative effect of day relative to laying on ln-transformed E2 concentrations (*p* < 0.001), indicating an overall decline as the reproductive period progressed. When time was grouped into consecutive 3-day reproductive windows, significant differences between windows were also detected, (*p* < 0.001). Estimated marginal means identified W−5 as the window with the highest E2 values, and Sidak-adjusted post hoc comparisons showed that W−5 was significantly higher than W−7 (*p* < 0.001), W−1 (*p* = 0.006), W0 (*p* < 0.001), and all post-laying windows from W+1 to W+6 (W+1: *p* = 0.002; W+2: *p* = 0.001; W+3: *p* < 0.001; W+4: *p* = 0.001; W+5: *p* = 0.002; W+6: *p* = 0.015). These results support a statistically significant pre-laying E2 peak around days −15 to −13, followed by lower concentrations around and after egg-laying.

#### 3.1.3. Integrated Temporal Pattern of Reproductive Hormones

**Figure 3:**
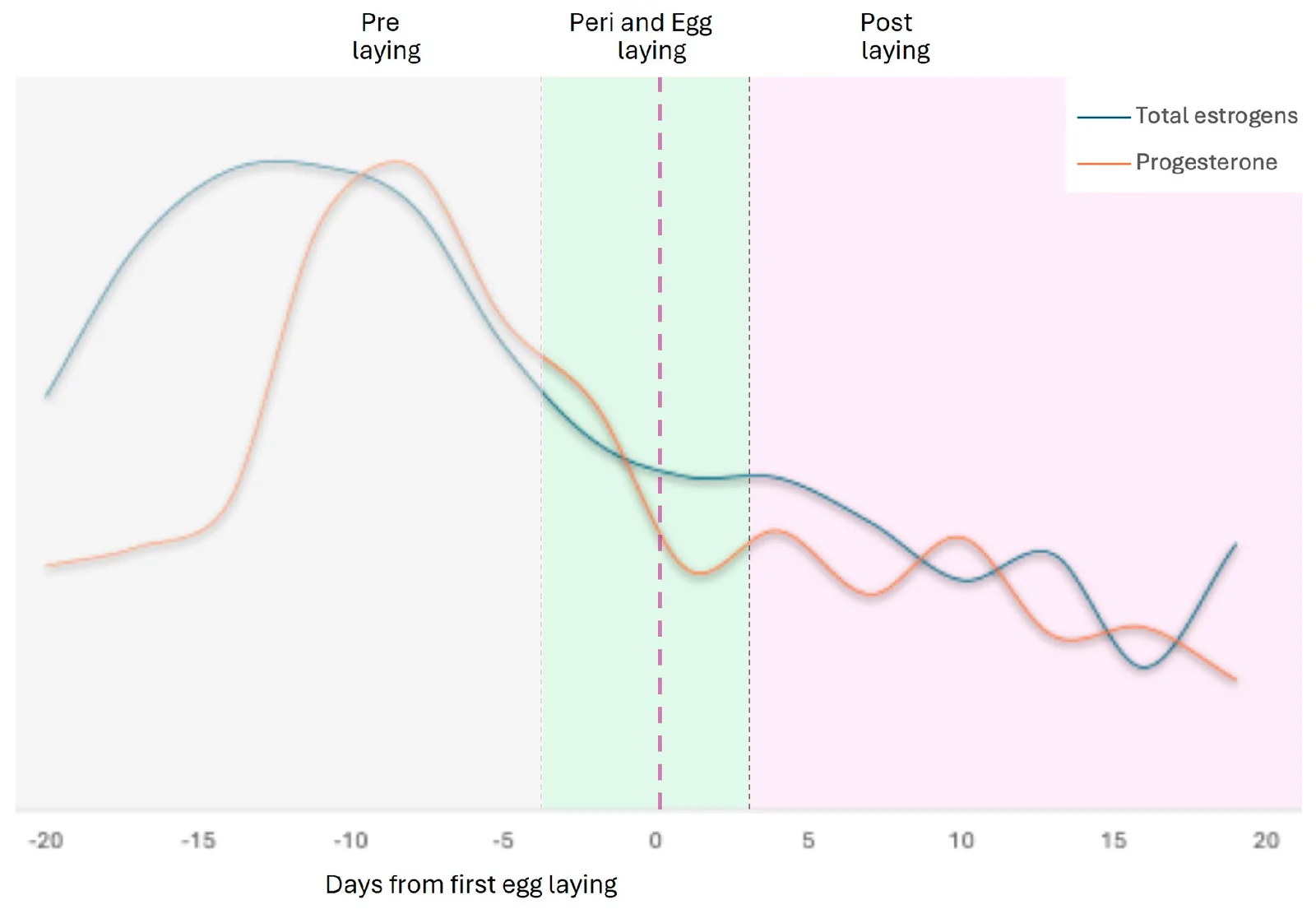
Integrated temporal pattern of salivary estrogens and progesterone in female gentoo penguins (*Pygoscelis papua*) during the peri-laying period. E1S and E2 were summed to describe the combined salivary estrogenic profile. Because estrogens and progesterone were measured in different units, values were normalized as percentage of the maximum concentration recorded for each hormone. Day 0 represents first egg-laying. Shaded areas indicate the reproductive phases: pre-laying, peri-laying, egg-laying, and post-laying.

For descriptive visualization of the reproductive hormone profiles, mean E1S and E2 concentrations were summed within each reproductive window to obtain a combined estrogenic profile. The combined estrogenic profile and mean P4 concentrations were then independently normalized and expressed as percentages of their respective maximum values across the study period. The normalized profiles showed a sequential pre-laying pattern: E2 reached its maximum in W−5, E1S in W−4, and P4 in W−3. The combined estrogenic profile therefore increased earlier than the progesterone profile, while both showed a decrease towards egg-laying and remained lower during the post-laying period (Figure 3). This integrated representation was used for descriptive comparison only, and no additional inferential analyses were performed on the normalized values.

### 3.2. Corticosterone and Testosterone Salivary Dynamics Linked to HPA-Axis Activity and Reproductive Behavior

#### 3.2.1. Corticosterone (CT)

**Figure 4:**
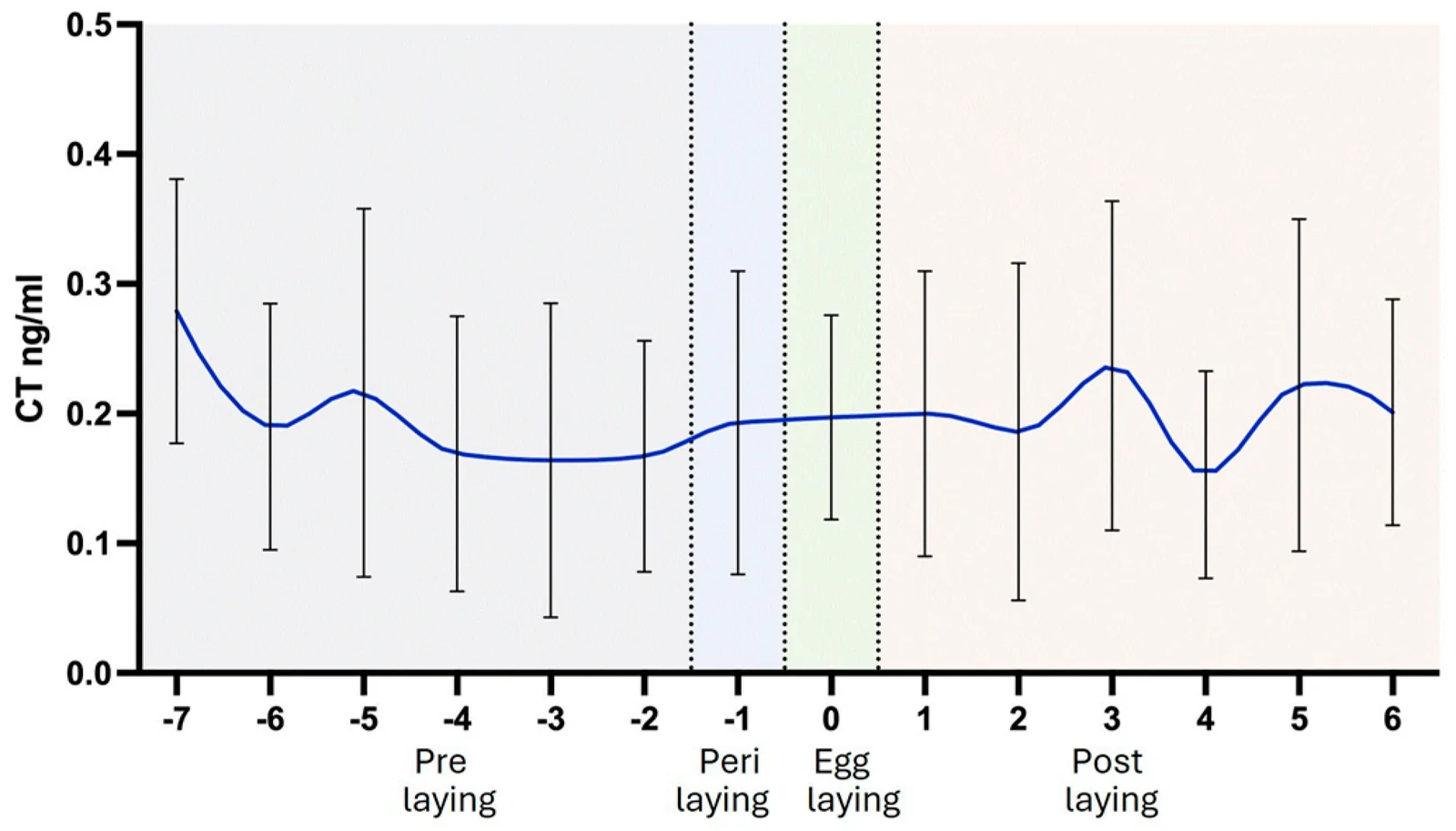
Temporal variation in salivary CT in female gentoo penguins (*Pygoscelis papua*) during the peri-laying period. Data are presented as mean ± SD for consecutive 3-day reproductive windows from W−7 to W+6. Day 0 represents first egg-laying. The profile shows a gradual decrease from the pre-laying to the post-laying period. Sample sizes by reproductive window were: W−7, *n* = 18; W−6, *n* = 19; W−5, *n* = 20; W−4, *n* = 19; W−3, *n* = 20; W−2, *n* = 17; W−1, *n* = 19; W0, *n* = 21; W+1, *n* = 18; W+2, *n* = 20; W+3, *n* = 17; W+4, *n* = 18; W+5, *n* = 19; and W+6, *n* = 18 (total *n* = 263).

The temporal profile of salivary CT concentrations is shown in Figure 4. A linear mixed model with random intercepts per individual and time relative to laying as a fixed effect was used to analyze the salivary CT. Considering day as a continuous covariate, the model showed a downward temporal trend across the study period in a logarithmic scale (*p* = 0.0289), with highest mean concentration of 0.279 ± 0.102 ng/mL at the beginning W−7 (−21 to −19 days) and a gradual decrease until reaching the minimum at W+4 (12 to 14 days), with values of 0.153 ± 0.080 ng/mL. However, when grouping time into consecutive 3-day windows, the overall contrast between windows was not significant (*p* = 0.1688).

#### 3.2.2. Testosterone (T)

**Figure 5:**
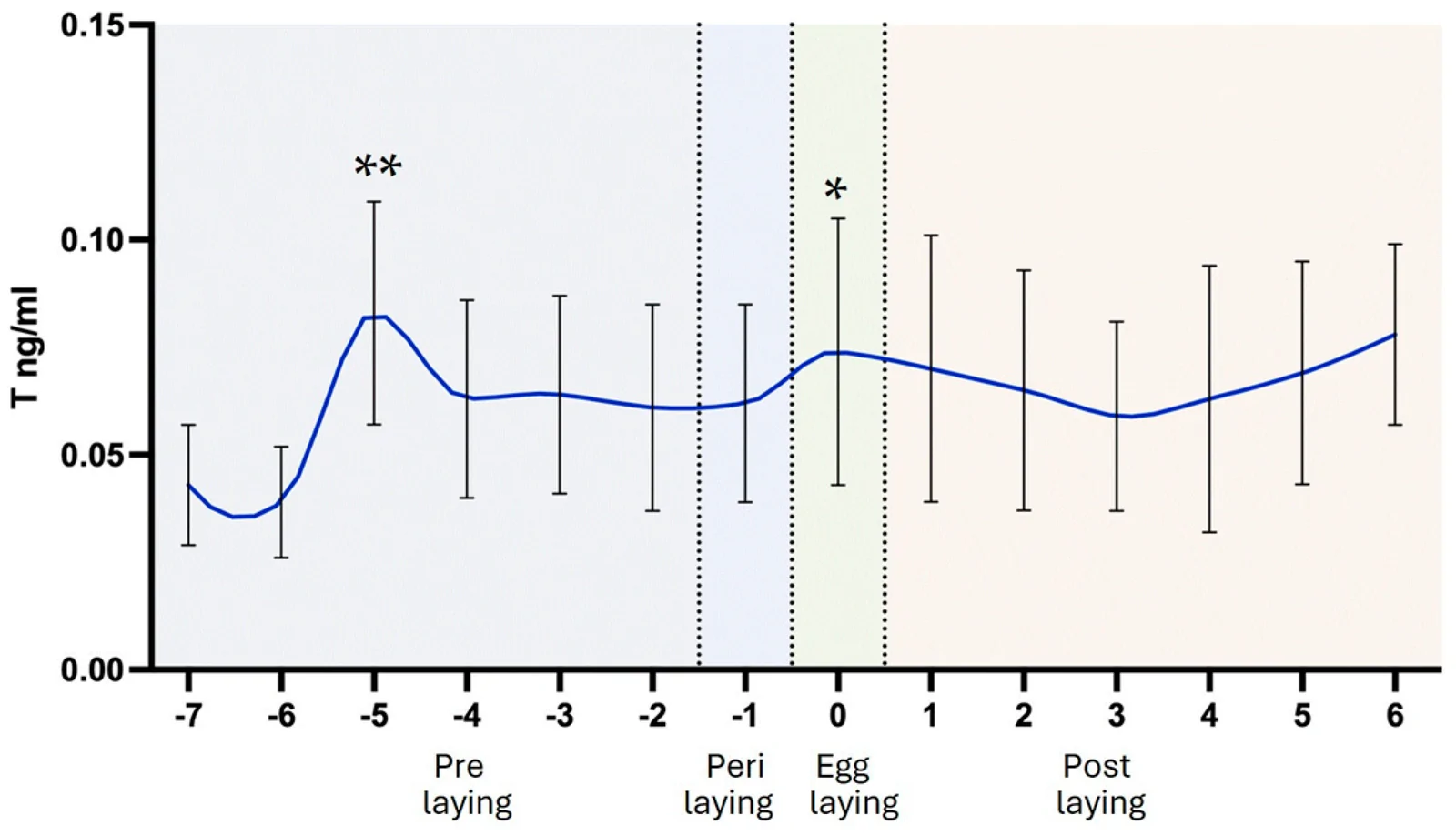
Temporal variation in salivary T in female gentoo penguins (*Pygoscelis papua*) during the reproductive period. Data are presented as mean ± SD for consecutive 3-day reproductive windows. Day 0 represents first egg-laying. Asterisks indicate significant Sidak-adjusted comparisons against W−6; * *p* < 0.05; ** *p* < 0.01. Sample sizes by reproductive window were: W−7, *n* = 18; W−6, *n* = 19; W−5, *n* = 20; W−4, *n* = 19; W−3, *n* = 20; W−2, *n* = 17; W−1, *n* = 19; W0, *n* = 21; W+1, *n* = 18; W+2, *n* = 20; W+3, *n* = 17; W+4, *n* = 18; W+5, *n* = 19; and W+6, *n* = 18 (total *n* = 263).

In the case of salivary T the temporal variation is shown in Figure 5; the highest individual value was recorded on day +2 (W0, 0.140 ng/mL), whereas the lowest value was observed on day +14 (W+4, 0.020 ng/mL). At the window level, the highest mean concentration was observed in W−5 (0.083 ± 0.026 ng/mL), while the lowest mean was observed in W−6 (0.039 ± 0.013 ng/mL).

Linear mixed models using ln-transformed concentrations and a random intercept for individual showed no significant temporal effect when day relative to laying was included as a continuous covariate (Type III test of fixed effects: *p* = 0.123). However, when time was grouped into consecutive 3-day windows, the overall contrast between windows was significant (Type III test of fixed effects: *p* = 0.006). Sidak-adjusted pairwise comparisons showed that testosterone concentrations were significantly higher in W−5 than in W−6 (*p* = 0.004; approximately 2.13-fold higher), and higher in W0 than in W−6 (*p* = 0.018; approximately 1.82-fold higher). These differences were concentrated in mid pre-laying (W−5) and around laying (W0) compared with the earlier pre-laying windows (W−7/W−6), rather than reflecting a progressive day-to-day trend.

## 4. Discussion

Unlike other non-invasive biological matrices used in penguins and other avian species, saliva may offer finer temporal resolution, allowing for the reflection of hormone concentrations close to the time of sampling as demonstrated in large-billed crows, where both serum and salivary corticosterone increased significantly within 10 min after an ACTH stimulation, although the salivary peak appeared to be delayed by approximately 30 min compared with the serum peak [33]. In the case of feces, fecal glucocorticoid metabolites have been used in penguins to assess their well-being or the impact of external factors [19,20,22], but they present the problem of accumulating data relative to hours or days and are subject to variability due to different influencing factors such as diet or environmental conditions [44,45]. Furthermore, they may present logistical limitations for their collection and individual identification in certain contexts such as large bird colonies, making their rapid preservation necessary to avoid or minimize the degradation of the samples [46]. In the case of feathers, the other non-invasive matrix used in studies with avian species, it allows the collection of corticosterone data throughout follicular growth, which can be a limiting factor in species that have an annual molt such as penguins [27,28]. For this reason, saliva is presented as a useful matrix for minimally invasive longitudinal studies, especially in facilities under human care where animal training can be performed.

The results obtained in our study reflect the validity of using saliva as a biological matrix for measuring steroid hormones (P4, E1S, E2, CT and T) in female gentoo penguins (*Pygoscelis papua*), allowing both the characterization of salivary corticosterone variation as a potential indicator of baseline HPA-axis activity and the monitoring of reproductive and social endocrine variation through progesterone, estrogens, and testosterone, expanding the taxonomic range in avian species of non-invasive monitoring of these hormones. The correlation observed between saliva and serum samples was high for all hormones with a correlation r^2^ ≥ 0.858, with testosterone showing the highest correlation at r^2^ = 0.990. It is important to note that the lower absolute concentrations obtained in saliva compared with serum were expected, since this matrix mainly reflects the free fraction not bound to proteins of circulating steroids [29]. These results are consistent with those obtained in other studies conducted on birds [33,34]. Specifically, the results of the study conducted on large-billed crows (*Corvus macrorhynchos*) observed parallel increases in serum and salivary corticosterone levels after an ACTH challenge and after handling. In the case of common gulls (*Larus canus*), the study obtained a moderate positive correlation between serum and saliva samples, reinforcing the usefulness of saliva in seabirds.

This advance in the use of saliva as a biological matrix for hormonal study may provide complementary information on HPA-axis activity in this penguin species with less invasive methodologies than those used to date, since blood extraction requires handling and immobilization of the animals. In penguins, handling and immobilization can produce rapid variations in baseline corticosterone levels, as has been demonstrated in both Adélie and emperor [17], in which the rise in corticosterone appeared within the first 5 min. Recently, it has been described that this response is also species and age dependent, with young penguins being more susceptible in the species of the genus *Pygoscelis* [18]. In our study, salivary corticosterone showed a gradual decrease throughout the study period rather than abrupt differences between specific reproductive windows, suggesting that salivary CT may reflect broader temporal changes rather than short-term peaks associated with particular phases. In this context, saliva offers an additional advantage. Experimental validations with birds have shown that salivary corticosterone increases with a latency of minutes relative to blood corticosterone, allowing values closer to baseline to be obtained even under field conditions with brief manipulations [33,34]. Following this approach, obtaining saliva voluntarily under desensitization or training, may reduce interference from the capture method and facilitates the collection of more robust data with less bias regarding the physiological state and actual baseline values of these species.

In our study, salivary progesterone (P4) reached a peak pre-laying value, with the maximum value occurring in the W−3 window (−9 to −7 days before laying), followed by a progressive decline toward and after laying. This progesterone pattern is consistent with its role in periovulatory physiology described in birds [47] and with published results in this and other penguin species showing progesterone increases in the periods preceding laying, although the window of peak values may vary between studies and penguin species [14,15,16]. These differences can be attributed to the matrix type and the temporal grouping of the samples.

In the case of estrogens, E2 and E1S showed a pre-laying increase, followed by a decrease around and after laying. E2 reached a peak during W−5 while E1S had it slightly later in W−4; this temporal displacement may reflect differences in hormone metabolism, conjugation and excretion dynamics. These data, along with those obtained for P4, are similar to what has been observed in birds in other studies, where estrogens increase in association with follicular growth and preceded the increase in progesterone in the preovulatory follicle, followed by a decrease in both values after laying [14,15,16]. The sequence of results obtained for E1S, E2 and P4 is consistent in the case of *P. papua*, with the possibility that ovulation could occur in a window after the P4 peak, probably around W−2, and that the egg remains in the oviduct for an extended period. This assessment agrees with studies in *S. magellanicus*, which suggest that ovulation occurs 98 h before laying [16]. This sequential pattern, summarized in Figure 3, is consistent with the progression from follicular growth and estrogenic activity to the pre-laying progesterone increase. To our knowledge, the plasma-to-saliva delay has not been quantified for reproductive steroids in avian species. In large-billed crows, serum and salivary corticosterone increased within 10 min after ACTH stimulation, although the maximum salivary concentration occurred approximately 30 min after the serum maximum [33], indicating that salivary concentrations may follow rapid systemic changes with a short, hormone- and species-dependent delay. However, this finding cannot be directly extrapolated to ovarian steroids. In Japanese quail, plasma E2, estrone, and testosterone peaked approximately 6 h before ovulation, whereas P4 peaked approximately 3 h before ovulation [48], demonstrating that systemic reproductive endocrine changes may precede ovulation by only a few hours.

The salivary corticosterone values showed a smooth temporal variation throughout the reproductive period analyzed. When using the model with day as a continuous covariate, a significant temporal effect was detected, but when grouping the data into windows like the rest of the hormones, no global differences were observed, which suggests that the change was progressive and likely influenced by high interindividual variability rather than by abrupt differences between specific reproductive phases. Descriptively, the values were highest in the pre-laying phase, with a maximum in the W−7, and decreased towards the post-laying period, with a minimum in the W+4. This pattern may reflect physiological modulation of adrenal activity associated with the reproductive period and the metabolic and endocrine adjustments preceding egg-laying [49]. It would be interesting to expand the study to include more physiological phases, such as molting, since it is a period of high energy demand and marked hormonal changes [38]. Due to the variation in glucocorticoids throughout the day, it would be relevant to study the diurnal profile of this species under polar conditions with continuous light, where the attenuation or absence of daily rhythms has been described in other species of the genus *Pygoscelis*, such as Adélie penguins [50].

The salivary T results did not show a consistent linear temporal trend across the reproductive period, although the window-based analysis detected localized differences, with higher concentrations in W−5 and W0 compared with W−6; this pattern may reflect episodic variation associated with reproductive physiology rather than a progressive endocrine change throughout the peri-laying period. In females, testosterone and other androgens participate in the ovarian steroidogenesis and can act as precursor for estrogen formation through aromatization. In avian follicles, steroid production is compartmentalized across follicular cell layers, supporting the role of androgens within the ovarian steroidogenic pathway [51]. The localized increase in −5 coinciding with the pre-laying estrogenic rise may represent a physiological component of follicular steroidogenesis rather than a direct periovulatory signal.

It has also been described in penguin females that testosterone may also be modulated in many cases by social contexts such as nest acquisition, defense and social competition, particularly during the early reproductive period [52]. Our results are consistent with the hypothesis that in *P. papua*, testosterone variations may provide complementary information on both ovarian activity and reproductive behavior. Previous studies have demonstrated an increase in testosterone in both sexes of *P. papua* during the postcopulatory period, suggesting that this is due to an association with nest defense behavior in both sexes [53]. These observations, along with those made in *P. adeliae*, suggest that testosterone in females of this genus may contribute to behaviors typical of the early stages of the reproductive cycle, including nest acquisition and subsequent defense, as well as social competition within the colony [54]. Future studies with more serial sampling and accompanied by behavioral assessments would help clarify the relative contribution of follicular steroidogenesis and reproductive behavior to salivary testosterone dynamics in this species.

From a zoological veterinary perspective, repeated voluntary saliva collection could provide complementary longitudinal endocrine information without requiring repeated restraint or venipuncture. Changes in salivary progesterone and estrogen profiles may help identify patterns compatible with ovarian activity and the approach to the peri-laying period, since changes in circulating reproductive steroids during the weeks preceding oviposition have been described in female penguins under human care [16], while pre-laying estrogen and progestogen metabolites increase have also been reported in zoo guans and cinereus vultures [55,56]. In females with a history of reproductive disorders or abnormal laying patterns, deviations from their individual endocrine profile could prompt closer surveillance and help determine the appropriate timing of clinical assessment and diagnostic imaging for suspected delayed oviposition, egg binding, or follicular dysfunction [57]. Salivary hormone profiles should be considered a complementary monitoring tool and interpreted together with behavioral observations, reproductive history, physical examination, and diagnostic imaging.

Among the limitations of this study, the sampling frequency of every 3 days may have reduced the ability to detect rapid changes in the concentrations of the hormones studied, which typically occur near ovulation. Although sampling occurred in an anticipatory feeding context, all swabs were collected before food intake, and no visible food particles or regurgitated material were observed. Nevertheless, subtle effects of feeding anticipation on salivary secretion cannot be completely excluded, and cotton-based collection devices may exhibit analyte-dependent steroid recovery. The use of identical standardized collection and processing procedures for all samples, together with the satisfactory analytical validation of the recovered salivary matrix, supports the longitudinal within-study comparisons. Future studies with more frequent sampling, behavioral assessments, and monitoring of the complete annual biological cycle of this species will allow for the establishment of specific baseline ranges and improve the applicability of the data for reproductive management and ex situ and in situ conservation of endangered species.

## 5. Conclusions

In conclusion, this study supports the use of saliva as a promising minimally invasive matrix for monitoring reproductive endocrine changes in female *P. papua*. Salivary progesterone, estradiol and estrone sulfate showed temporal changes during the reproductive period, while corticosterone and testosterone provided additional information on physiological and behavioral variations around laying. Although salivary hormone monitoring in our study is constrained by the 3-day sampling interval, and salivary hormone measurements may be influenced by biological variability, residual matrix effects, and assay-specific factors, the validation results obtained in this study support its usefulness as a complementary approach for repeated endocrine assessment. The ability to obtain voluntary and repeated samples may provide a complementary tool for reproductive monitoring in zoological institutions. Following application-specific validation and integration with behavioral, clinical and reproductive data could contribute to future welfare-oriented assessments and conservation breeding programs, two fundamental aspects of 21st-century zoological institutions.

## Figures and Tables

**Table 1:** Analytical validation parameters for salivary hormone assays in female gentoo penguins. Serum–saliva associations were calculated from eight independent paired observations, comprising one simultaneously collected serum and saliva sample per female.

Hormone	Recovery (%)	Intra-Assay CV (%)	Inter-Assay CV (%)	Serum–Saliva r^2^	*p*-Value
P4	96.14 ± 7.18	7.08 ± 1.62	7.48 ± 0.26	0.910	<0.001
E1S	91.20 ± 3.45	7.54 ± 1.04	6.83 ± 0.55	0.858	<0.001
E2	97.96 ± 4.98	7.07 ± 0.56	7.91 ± 0.22	0.879	<0.001
CT	92.05 ± 9.26	5.36 ± 0.42	6.76 ± 0.71	0.919	<0.001
T	90.89 ± 9.36	3.07 ± 0.25	6.54 ± 1.17	0.990	<0.001

## Data Availability

The data that support the findings of this study are available from the corresponding author upon reasonable request.

## Abbreviations

The following abbreviations are used in this manuscript:

P4	Progesterone
E1S	Estrone sulphate
E2	17β-estradiol
CT	Corticosterone
T	Testosterone

## References

[1] Kersey D.C., Dehnhard M. (2014). The use of noninvasive and minimally invasive methods in endocrinology for threatened mammalian species conservation. Gen. Comp. Endocrinol..

[2] Ganswindt A., Brown J.L., Freeman E.W., Kouba A.J., Penfold L.M., Santymire R.M., Vick M.M., Wielebnowski N., Willis E.L., Milnes M.R. (2012). International Society for Wildlife Endocrinology: The future of endocrine measures for reproductive science, animal welfare and conservation biology. Biol. Lett..

[3] Schilling A.-K., Mazzamuto M.V., Romeo C. (2022). A Review of Non-Invasive Sampling in Wildlife Disease and Health Research: What’s New?. Animals.

[4] Boersma P.D. (2008). Penguins as marine sentinels. BioScience.

[5] Weichler T., Garthe S., Luna-Jorquera G., Moraga J., Thiel M. (2004). Seabird distribution on the Humboldt Current in northern Chile in relation to hydrography, productivity and fisheries. ICES J. Mar. Sci..

[6] IUCN (2024). The IUCN Red List of Threatened Species.

[7] Trathan P.N., García-Borboroglu P., Boersma D., Bost C.A., Crawford R.J., Crossin G.T., Cuthbert R.J., Dann P., Davis L.S., De la Puente S. (2015). Pollution, habitat loss, fishing, and climate change as critical threats to penguins. Conserv. Biol..

[8] Gimeno M., Giménez J., Chiaradia A., Davis L.S., Seddon P.J., Ropert-Coudert Y., Reisinger R.R., Coll M., Ramírez F. (2024). Climate and human stressors on global penguin hotspots: Current assessments for future conservation. Glob. Change Biol..

[9] Puasa N.A., Zulkharnain A., Verasoundarapandian G., Wong C.Y., Zahri K.N.M., Merican F., Shaharuddin N.A., Gomez-Fuentes C., Ahmad S.A. (2021). Effects of diesel, heavy metals and plastics pollution on penguins in Antarctica: A review. Animals.

[10] Boersma P.D., Rebstock G.A. (2014). Climate change increases reproductive failure in Magellanic penguins. PLoS ONE.

[11] García Borboroglu P., Boersma P.D. (2013). Penguins: Natural History and Conservation.

[12] Ainley D.G., Wilson R.P. (2023). The Aquatic World of Penguins: Biology of Fish-Birds.

[13] Juarez Martinez I., Kacelnik A., Jones F.M., Hinke J.T., Dunn M.J., Raya Rey A., Lynch H.J., Owen K., Hart T. (2026). Record phenological responses to climate change in three sympatric penguin species. J. Anim. Ecol..

[14] Williams T.D. (1992). Reproductive endocrinology of macaroni (*Eudyptes chrysolophus*) and gentoo (*Pygoscelis papua*) penguins. II. Plasma levels of gonadal steroids and LH in immature birds in relation to deferred sexual maturity. Gen. Comp. Endocrinol..

[15] Groscolas R., Jallageas M., Goldsmith A., Assenmacher I. (1986). The endocrine control of reproduction and molt in male and female emperor (*Aptenodytes forsteri*) and Adelie (*Pygoscelis adeliae*) penguins. I. Annual changes in plasma levels of gonadal steroids and LH. Gen. Comp. Endocrinol..

[16] O’Brien J.K., Schmitt T.L., Nollens H.H., Dubach J.M., Robeck T.R. (2016). Reproductive physiology of the female Magellanic penguin (*Spheniscus magellanicus*): Insights from the study of a zoological colony. Gen. Comp. Endocrinol..

[17] Cockrem J.F., Potter M.A., Barrett D.P., Candy E.J. (2008). Corticosterone responses to capture and restraint in emperor and Adelie penguins in Antarctica. Zool. Sci..

[18] Machado Pinto J.A., Souza-Kasprzyk J., Padilha J.A.G., Viau P., Torres J.P.M., Alves M.A.S., Costa E.S. (2025). Comparative corticosterone responses to capture stress in sympatric Antarctic penguins. Gen. Comp. Endocrinol..

[19] Xie S., McWhorter T.J. (2021). Fecal glucocorticoid metabolite concentration as a tool for assessing impacts of interventions in Humboldt penguins (*Spheniscus humboldti*). Birds.

[20] Itoh M., Kitahara M., Sawayama N., Matsumoto N., Toyotome T., Yamada K. (2024). Evaluation of the stress state based on fecal corticosterone metabolite concentrations in captive penguins in Japan. J. Vet. Med. Sci..

[21] Ninnes C.E., Waas J.R., Ling N., Nakagawa S., Banks J.C., Bell D.G., Bright A., Carey P.W., Chandler J., Hudson Q.J. (2010). Comparing plasma and faecal measures of steroid hormones in Adelie penguins (*Pygoscelis adeliae*). J. Comp. Physiol. B.

[22] Lynch M.A., Youngflesh C., Agha N.H., Ottinger A.M., Lynch H.J. (2019). Tourism and stress hormone measures in gentoo penguins on the Antarctic Peninsula. Polar Biol..

[23] Will A., Watanuki Y., Kikuchi D.M., Sato N., Ito M., Callahan M., Wynne-Edwards K., Hatch S., Elliott K., Slater L. (2015). Feather corticosterone reveals stress associated with dietary changes in a breeding seabird. Ecol. Evol..

[24] Voit M., Merle R., Baumgartner K., von Fersen L., Reese L., Ladwig-Wiegard M., Will H., Tallo-Parra O., Carbajal A., Lopez-Bejar M. (2020). Validation of an alternative feather sampling method to measure corticosterone. Animals.

[25] Reese L., Baumgartner K., von Fersen L., Merle R., Ladwig-Wiegard M., Will H., Haase G., Tallo-Parra O., Carbajal A., Lopez-Bejar M. (2020). Feather corticosterone measurements of greater flamingos living under different forms of flight restraint. Animals.

[26] Romero L.M., Fairhurst G.D. (2016). Measuring corticosterone in feathers: Strengths, limitations, and suggestions for the future. Comp. Biochem. Physiol. A Mol. Integr. Physiol..

[27] Lewden A., Halna du Fretay T., Stier A. (2024). Changes in body surface temperature reveal the thermal challenge associated with catastrophic moult in captive gentoo penguins. J. Exp. Biol..

[28] Wells N.C.A., Ledwidge M.J., Dann P., Walker M.J., Arnould J.P.Y. (2025). Mass loss, timing and duration of catastrophic moult in little penguins. Biol. Open.

[29] Vining R.F., McGinley R.A. (1987). The measurement of hormones in saliva: Possibilities and pitfalls. J. Steroid Biochem..

[30] Amaral R.S. (2010). Use of alternative matrices to monitor steroid hormones in aquatic mammals: A review. Aquat. Mamm..

[31] Amaral R.S., Rosas F.C., da Silva V.M., Nichi M., Oliveira C.A. (2013). Endocrine monitoring of the ovarian cycle in captive female Amazonian manatees (*Trichechus inunguis*). Anim. Reprod. Sci..

[32] Illera J.C., Silván G., Cáceres S., Carbonell M.D., Gerique C., Martínez-Fernández L., Munro C., Casares M. (2014). Assessment of ovarian cycles in the African elephant (*Loxodonta africana*) by measurement of salivary progesterone metabolites. Zoo Biol..

[33] Aota I., Yatsuda C., Izawa E.I. (2023). Salivary corticosterone measurement in large-billed crows by enzyme-linked immunosorbent assay. J. Vet. Med. Sci..

[34] Carbillet J., Saks L., Sepp T. (2024). Salivary corticosterone reflects plasmatic levels in a wild seabird. Gen. Comp. Endocrinol..

[35] Festing M.F.W. (2018). On determining sample size in experiments involving laboratory animals. Lab. Anim..

[36] AZA Penguin Taxon Advisory Group (2014). Penguin (Spheniscidae) Care Manual.

[37] Ancel A., Beaulieu M., Gilbert C. (2013). The different breeding strategies of penguins: A review. Comptes Rendus Biol..

[38] Lescroël A., Bajzak C., Bost C.-A. (2009). Breeding ecology of the gentoo penguin Pygoscelis papua at Kerguelen Archipelago. Polar Biol..

[39] Mauget R., Garcia V., Jouventin P. (1995). Endocrine basis of the reproductive pattern of the Gentoo penguin (*Pygoscelis papua*): Winter breeding and extended laying period in northern populations. Gen. Comp. Endocrinol..

[40] Redrobe S.P. (2020). Blood sampling techniques in penguins. Proceedings of the International Association of Aquatic Animal Medicine (IAAAM).

[41] Illera J.C., Silván G., Munro C.J., Lorenzo P.L., Illera M.J., Liu I.K.M., Illera M. (2003). Amplified androstenedione enzymeimmunoassay for the diagnosis of cryptorchidism in the male horse: Comparison with testosterone and estrone sulphate methods. J. Steroid Biochem. Mol. Biol..

[42] Abraham G.E., Cameron E.H.D., Hillier S.G., Griffiths K. (1975). Characterization of anti-steroid antisera. Steroid Immunoassay: Proceedings of the Fifth Tenovus Workshop, Cardiff, April 1974.

[43] Munro C., Lasley B. (1987). Non-radiometric methods for immunoassay of steroid hormones. Prog. Clin. Biol. Res..

[44] Palme R. (2019). Non-invasive measurement of glucocorticoids: Advances and problems. Physiol. Behav..

[45] Goymann W. (2012). On the use of non-invasive hormone research in uncontrolled, natural environments: The problem with sex, diet, metabolic rate and the individual. Methods Ecol. Evol..

[46] Carbillet J., Palme R., Maublanc M.-L., Cebe N., Gilot-Fromont E., Verheyden H., Rey B. (2023). Instability of fecal glucocorticoid metabolites at 4 °C: Time to freeze matters. J. Exp. Zool. A Ecol. Integr. Physiol..

[47] Gil D., Brockmann H.J., Roper T.J., Naguib M., Wynne-Edwards K.E., Barnard C., Mitani J.C. (2008). Hormones in Avian Eggs: Physiology, Ecology and Behavior. Advances in the Study of Behavior.

[48] Gulati D.P., Nakamura T., Tanabe Y. (1981). Diurnal variations in plasma LH, progesterone, testosterone, estradiol, and estrone in the Japanese quail. Poult. Sci..

[49] Deviche P., Bittner S., Gao S., Valle S. (2017). Roles and Mechanistic Bases of Glucocorticoid Regulation of Avian Reproduction. Integr. Comp. Biol..

[50] Vleck C.M., Van Hook J.A. (2002). Absence of daily rhythms of prolactin and corticosterone in Adélie penguins under continuous daylight. Condor.

[51] Porter T.E., Hargis B.M., Silsby J.L., El Halawani M.E. (1989). Differential Steroid Production between Theca Interna and Theca Externa Cells: A Three-Cell Model for Follicular Steroidogenesis in Avian Species. Endocrinology.

[52] Ketterson E.D., Nolan V., Sandell M. (2005). Testosterone in females: Mediator of adaptive traits, constraint on sexual dimorphism, or both?. Am. Nat..

[53] Williams T.D. (1992). Reproductive endocrinology of macaroni (*Eudyptes chrysolophus*) and gentoo (*Pygoscelis papua*) penguins. I. Seasonal changes in plasma levels of gonadal steroids and LH in breeding adults. Gen. Comp. Endocrinol..

[54] Vleck C.M., Bucher T.L., Reed W.L., Kristmundsdottir A.Y., Adams N.J., Slotow R.H. (1999). Changes in reproductive hormones and body mass through the reproductive cycle in the Adélie Penguin (*Pygoscelis adeliae*), with associated data on courting-only individuals. Proceedings of the 22nd International Ornithological Congress.

[55] Kozlowski C.P., Clawitter H.L., Asa C.S., Macek M.S., Snyder T.L., Tieber A.M. (2018). Patterns of fecal steroids associated with reproduction in two Cracidae species: The blue-throated piping guan (*Pipile cumanensis cumanensis*) and the horned guan (Oreophasis derbianus). J. Zoo Aquar. Res..

[56] Kozlowski C.P., Clawitter H., Fischer F., Bender A., Tieber A., Franklin A., Willis M.J., Powell D. (2023). Patterns of faecal hormone metabolites associated with reproduction in cinereous vultures Aegypius monachus. J. Zoo Aquar. Res..

[57] Abou-Zahr T., Calvo Carrasco D., Jones S.L., Dutton T.A.G. (2019). Percloacal ovocentesis in the treatment of avian egg binding: Review of 20 cases. J. Avian Med. Surg..

